# Genetic Analysis of *Vibrio parahaemolyticus* O3:K6 Strains That Have Been Isolated in Mexico Since 1998

**DOI:** 10.1371/journal.pone.0169722

**Published:** 2017-01-18

**Authors:** Abraham Guerrero, Marcial Leonardo Lizárraga-Partida, Bruno Gómez Gil Rodríguez, Alexei Fedorovish Licea-Navarro, Valeria Jeanette Revilla-Castellanos, Irma Wong-Chang, Ricardo González-Sánchez

**Affiliations:** 1 Centro de Investigación Científica y de Educación Superior de Ensenada, Baja California, (CICESE), Ensenada, Baja California, México; 2 Mazatlan Unit for Aquaculture and Environmental Management, (CIAD, AC), Mazatlán, Sinaloa, México; 3 Instituto de Ciencias del Mar y Limnología, Universidad Nacional Autónoma de México (ICMyL/UNAM), México D.F., México; Beijing Institute of Microbiology and Epidemiology, CHINA

## Abstract

*Vibrio parahaemolyticus* is an important human pathogen that has been isolated worldwide from clinical cases, most of which have been associated with seafood consumption. Environmental and clinical toxigenic strains of *V*. *parahaemolyticus* that were isolated in Mexico from 1998 to 2012, including those from the only outbreak that has been reported in this country, were characterized genetically to assess the presence of the O3:K6 pandemic clone, and their genetic relationship to strains that are related to the pandemic clonal complex (CC3). Pathogenic *tdh*^+^ and *tdh*^+^/*trh*^+^ strains were analyzed by pulsed-field gel electrophoresis (PFGE) and multilocus sequence typing (MLST). Also, the entire genome of a Mexican O3:K6 strain was sequenced. Most of the strains were *tdh*/ORF8-positive and corresponded to the O3:K6 serotype. By PFGE and MLST, there was very close genetic relationship between ORF8/O3:K6 strains, and very high genetic diversities from non-pandemic strains. The genetic relationship is very close among O3:K6 strains that were isolated in Mexico and sequences that were available for strains in the CC3, based on the PubMLST database. The whole-genome sequence of CICESE-170 strain had high similarity with that of the reference RIMD 2210633 strain, and harbored 7 pathogenicity islands, including the 4 that denote O3:K6 pandemic strains. These results indicate that pandemic strains that have been isolated in Mexico show very close genetic relationship among them and with those isolated worldwide.

## Introduction

*Vibrio parahaemolyticus* is a bacterium that inhabits warm marine environments and are able to causes gastroenteritis, wound infections and septicemia, that are associated with the ingestion of raw seafood, primarily filter-feeding bivalves mollusks [1]. The pathogenicity of this bacterium has been correlated with several virulent factors, including thermostable direct hemolysin (TDH), TDH-related hemolysin (TRH) and the type III secretion system (T3SS) [2–5]. *V*. *parahaemolyticus* strains that contain *tdh* or *trh*, are considered to be pathogenic [6].

The first clinical cases of *V*. *parahaemolyticus* were reported in Japan in 1950 [7], although in 1996, an atypically rise in infections occurred in India due to *tdh*^+^/O3:K6 isolates [8], spreading to southeast Asia in just a few months [9]. Subsequently, this clone has been associated with most *V*. *parahaemolyticus* clinical cases worldwide [10–12]. Genetic studies have demonstrated that all the post-1996 O3:K6 strains, are identical but differ significantly from earlier isolates, also known as old-O3:K6 (*tdh*^*-*^*/trh*^+^) strains [13]. Post-1996 *tdh*^*+*^/O3:K6 strains have the same MLST sequence type (ST-3) and are considered to be the ancestors of the pandemic serovariants, which now constitute the pandemic clonal complex (CC3) [14].

In the Americas, the pandemic strain O3:K6 was first isolated from a clinical case in Peru in 1996 [15], after which outbreaks of this strain were reported in Peru in 1998 [15]; Chile in 1997,1998, 2004 and 2005 [16]; the US in 1998 [17] and Brazil in 2002 [18]. In Mexico, 1 localized outbreak (2003 to 2004) of *V*. *parahaemolyticus* O3:K6 strains was reported on its northwest Pacific coast, where more than 1200 clinical cases were registered after ingestion of raw shrimps [19]. Velazquez-Roman *et al*. [20], isolated the pandemic clone O3:K6 from clinical and environmental sources, in the northwest state of Sinaloa, Mexico from 2004 to 2010. Recently, Hernández-Díaz *et al*. [21] indicated that the pandemic clone O3:K6 has established itself endemically in the northwest Pacific coast of Mexico.

The surveillance programs that have been implemented by the Mexican Diagnostics and Epidemiological Reference Institute (InDRE), since 1991 focus on pathogenic vibrios, especially toxigenic *V*. *cholerae* [22]. Nevertheless, suspicious strains of *V*. *parahaemolyticus* have been isolated and maintained in the InDRE strain collection and donated to CICESE for future studies. These strains, those isolated in 2004 during the outbreak in Mexico, and environmental strains that have been isolated from shrimps, oysters and biofouling of ships hulls, were examined in this study (Table 1).

**Table 1:** *Vibrio parahaemolyticus* strains from CICESE and CAIM culture collections.

Strain	Collection origin	Year	Source	Location	*tdh*	*trh*	orf8	Serotype
CAIM 728*	**T3979**	**1951**	**Clinical**	**Jap**	**+**	**-**	**-**	**O5:K12**
**CAIM 729**^T^*	**TX2103**^**T**^	**1998**	**Clinical**	**Tex**	**+**	**-**	**+**	**O3:K6**
**CAIM 1400**^T^*	**CAIM**	**2004**	**Clinical**	**Sin**	**+**	**-**	**+**	**O3:K6**
CAIM 1435*	**CAIM**	**2004**	**Clinical**	**Sin**	**+**	**-**	**-**	**NA**
CAIM 1474*	**CAIM**	**2004**	**Clinical**	**Sin**	**+**	**-**	**+**	**O3:K6**
CAIM 1477*	**CAIM**	**2004**	**Clinical**	**Sin**	**+**	**-**	**+**	**O3:K6**
CAIM 1490*	**CAIM**	**2004**	**Clinical**	**Sin**	**+**	**-**	**+**	**O3:K6**
CAIM 1693*	**CAIM**	**2004**	**Clinical**	**Sin**	**+**	**-**	**+**	**O3:K6**
**CAIM 1772**	**CAIM**	**2005**	**Shrimp**	**Sin**	**+**	**+**	**-**	**O5:K17**
CICESE-170*	**InDRE**	**1998**	**Clinical**	**Hgo**	**+**	**-**	**+**	**O3:K6**
CICESE-171*	**InDRE**	**1998**	**Clinical**	**Hgo**	**+**	**-**	**+**	**O3:K6**
CICESE-172*	**InDRE**	**1998**	**Clinical**	**CDMX**	**+**	**-**	**-**	**NA**
CICESE-173*	**InDRE**	**1998**	**Clinical**	**Hgo**	**+**	**-**	**+**	**O3:K6**
CICESE-174*	**InDRE**	**1998**	**Clinical**	**CDMX**	**+**	**-**	**+**	**O3:K6**
CICESE-175*	**InDRE**	**1998**	**Clinical**	**Hgo**	**+**	**-**	**+**	**O3:K6**
CICESE-176*	**InDRE**	**1998**	**Clinical**	**Hgo**	**+**	**-**	**+**	**O3:K6**
CICESE-177*	**InDRE**	**1998**	**Clinical**	**Mich**	**+**	**-**	**+**	**O3:K6**
CICESE-178*	**InDRE**	**1998**	**Clinical**	**Hgo**	**+**	**-**	**+**	**O3:K6**
CICESE-179*	**InDRE**	**1998**	**Clinical**	**Hgo**	**+**	**-**	**+**	**O3:K6**
CICESE-180*	**InDRE**	**1998**	**Clinical**	**Hgo**	**+**	**-**	**+**	**O3:K6**
CICESE-181*	**InDRE**	**1998**	**Clinical**	**Hgo**	**+**	**-**	**+**	**O3:K6**
CICESE-182*	**InDRE**	**1998**	**Clinical**	**Hgo**	**+**	**-**	**+**	**O3:K6**
CICESE-183*	**InDRE**	**1998**	**Clinical**	**Hgo**	**+**	**-**	**+**	**O3:K6**
CICESE-184*	**InDRE**	**1998**	**Clinical**	**Hgo**	**+**	**-**	**+**	**O3:K6**
CICESE-185*	**InDRE**	**1998**	**Clinical**	**Hgo**	**+**	**-**	**+**	**O3:K6**
CICESE-186*	**InDRE**	**1999**	**Clinical**	**Hgo**	**+**	**-**	**+**	**O3:K6**
CICESE-187*	**InDRE**	**2000**	**Clinical**	**Tamps**	**+**	**-**	**+**	**O3:K6**
CICESE-188*	**InDRE**	**2009**	**Clinical**	**NL**	**+**	**-**	**+**	**O3:K6**
CICESE-250*	**CICESE**	**2012**	**Biofouling**	**BC**	**+**	**-**	**-**	**NA**
CICESE-251*	**CICESE**	**2012**	**Biofouling**	**BC**	**+**	**-**	**-**	**NA**
**CICESE-253**	**CICESE**	**2012**	**Biofouling**	**BC**	**+**	**-**	**-**	**NA**
**CICESE-254**	**CICESE**	**2012**	**Biofouling**	**BC**	**+**	**-**	**-**	**NA**
CICESE-273*	**CICESE**	**2012**	**Biofouling**	**BC**	**+**	**-**	**+**	**O3:K6**
**CICESE-274**	**CICESE**	**2012**	**Biofouling**	**BC**	**+**	**-**	**-**	**NA**
CICESE-374*	**CICESE**	**2011**	**Oyster**	**CDMX**	**+**	**+**	**-**	**NA**
CICESE-375*	**CICESE**	**2011**	**Oyster**	**CDMX**	**+**	**+**	**-**	**NA**
**CICESE-376**	**CICESE**	**2011**	**Oyster**	**CDMX**	**+**	**+**	**-**	**NA**
**CICESE-390**	**CICESE**	**2011**	**Oyster**	**CDMX**	**+**	**+**	**-**	**NA**

(Jap) Japan, (Tex) Texas, USA), Mexican states: Sin (Sinaloa), Hgo (Hidalgo) CDMX (Ciudad de México), Mich (Michoacán), Tamps (Tamaulipas), NL (Nuevo León).

(*) Strains selected for MLST analysis. NA: not analyzed.

The aim of this study was to evaluate the presence of pandemic *V*. *parahaemolyticus* O3:K6 strains in Mexico, isolated from the environment and clinical cases, from 1998 to 2012 and determine their similarity with O3:K6 strains that have been isolated worldwide. Our results confirm the presence of the pandemic strain since 1998.

## Material and Methods

### Bacterial isolates

Thirty-eighth toxigenic *V*. *parahaemolyticus* strains were selected. Nineteen clinical strains were isolated by the InDRE from stool samples in hospitals from various Mexican states and years. Nine strains were obtained from the Collection of Aquatic Important Microorganisms (www.ciad.mx/caim), including strain CAIM 729 (TX2103^T^), which was isolated during the 1998 Texas (USA) outbreak [23,24], and strain CAIM 728, isolated in Japan. Most CAIM strains were isolated in 2004 during the outbreak in Mexico [19]. Four strains were isolated from oysters (*Crassostrea* spp.) that were collected at the La Nueva Viga seafood market in Mexico City in 2011, and 6 strains were isolated from the biofouling of commercial ships hulls in 2012 [25]. Strains that were isolated from oysters were examined per the Bacteriological Analytical Manual [26]. Strains were identified as *V*. *parahaemolyticus*, using the species-specific molecular markers *tlh* [27] and pR72H [28], homolysins were detected with *tdh* and *trh* specific primers [27]. Only those strains that were positive for the *tdh* and/or *trh* hemolysin genes were included (Table 1).

### DNA extraction and PCR amplification

Genomic DNA from overnight Luria-Bertani (LB) broth cultures of suspicious *V*. *parahaemolyticus* strains was purified with the Wizard^TM^ Genomic Kit (Promega) per the manufacturer. Purified DNA was diluted in RNAse/DNAse-free water (~50 ng μl^-1^). The strains were tested by PCR for presence of the pR72H, *tlh*, *tdh*, *trh* and ORF8 genes. The PCR was performed using the conditions and primers as reported in the literature (Table 2). The PCR products were amplified with GoTaq^TM^ DNA polymerase (Promega), and amplicons were resolved by gel electrophoresis (1.5% agarose) in 1X TBE (45 mM Tris, 45 mM boric acid, 10 mM EDTA, pH 8.0) and visualized under UV light. The strains CAIM 1400^T^ (*tdh*^+^/ORF8^+^) and CAIM 1772 (*tdh*^+^/*trh*^+^) were used as controls for the PCR analysis.

**Table 2:** Primers used for the molecular characterization of *V*. *parahaemolyticus*.

*Gene*	Primer	Amplicon	Sequence size	Reference
*tlh*	tlh-F AAAGCGGATTATGCAGAAGCACTG	450	-	27
	tlh-R GCTACTTTCTAGCATTTTCTCTGC			
*pR72H*	VP33-F TGCGAATTCGATAGGGTGTTAACC	387	-	28
	VP32-R CGAATCCTTGAACATACGCAGC			
*trh*	trh-F TTGGCTTCGATATTTTCAGTATCT	500	-	27
	trh-R CATAACAAACATATGCCCATTTCCG			
*tdh*	tdh-F GTAAAGGTCTCTGACTTTTGGAC	269	-	27
	tdh-R TGGAATAGAACCTTCATCTTCACC			
*ORF8*	O3MM824-F AGGACGCAGTTACGCTTGATG	369	-	24
	O3MM1192-R CTAACGCATTGTCCCTTTGTAG			
	MLST			
	CHROMOSOME I			
*dnaE*	dnaE-F tgtaaaacgacggccagtCGRATMACCGCTTTCGCCG	596	557	PubMLST
	dnaE-R caggaaacagctatgaccGAKATGTGTGAGCTGTTTGC			
*gyrB*	gyrB-F tgtaaaacgacggccagtGAAGGBGGTATTCAAGC	629	592	PubMLST
	gyrB-R caggaaacagctatgaccGAGTCACCCTCCACWATGTA			
*recA*	recA-F CGCCGCTGCGCTAGGTCAAA	773	731	This study
	recA-R CGCGATTTCTGGATGCTCACGC			
	CHROMOSOME II			
*dtdS*	dtdS-F tgtaaaacgacggccagtTGGCCATAACGACATTCTGA	497	458	PubMLST
	dtdS-R caggaaacagctatgaccGAGCACCAACGTGTTTAGC			
*pntA*	pntA-F- GGTGCGGAGTTCCTGACCGTG	470	430	This study
	pntA-R AACGCAGCAGGTGCTACCGA			
*pyrC*	pyrC-F tgtaaaacgacggccagtAGCAACCGGTAAAATTGTCG	533	493	PubMLST
	pyrC-R caggaaacagctatgaccCAGTGTAAGAACCGGCACAA			
*tnaA*	tnaA-F tgtaaaacgacggccagtTGTACGAAATTGCCACCAAA	463	423	PubMLST
	tnaA-R caggaaacagctatgaccAATATTTTCGCCGCATCAAC			

### Serotyping

A commercial *V*. *parahaemolyticus* antiserum kit (Denka Seiken, Tokyo, Japan), was used for serological typing per the manufacturer’s instructions (www.abcam.com/technical). CAIM 1400^T^ was used as a positive control for O3:K6, and CAIM 1772 (O5:K17), was included as a negative control.

### PFGE analysis

Thirty-eight *V*. *parahaemolyticus* and 2 *V*. *cholerae* strains (Table 1), were analyzed by PFGE with the standardized CDC protocol for *V*. *cholerae* [29], with minor modifications. Briefly, genomic DNA was digested with 20 U of Not-I (New England BioLabs), and restriction fragments were resolved on a CHEF Mapper^TM^ PFGE system (BioRad). The run time was divided into 2 blocks, 13 h (2–10-s pulse time) and 12 h (20–25-s pulse time) at 6 V cm^-1^ with an angle of 120°. Lambda ladder for PFGE (New England BioLabs) was used as the molecular size marker. Thiourea (50 μM) was added during the electrophoresis. Gels were stained with ethidium bromide and visualized under UV light. Images of the gels were taken after electrophoresis and were used to generate a dendrogram with GelCompar II (Applied Maths, Kortrijk, Belgium) by Dice coefficient-unweighted pair group method with arithmetic averages (UPGMAs).

### MLST analysis

Thirty-two strains that were classified as *tdh*^*+*^ and *tdh*^*+*^/*trh*^*+*^ were selected from the PFGE clusters (Table 1) for analysis by MLST. Seven loci from both chromosomes (Table 2) were chosen; 5 loci were amplified using primers in the PubMLST database for *V*. *parahaemolyticus* (http://pubmlst.org/vparahaemolyticus/) by PCR per González-Escalona *et al*. [14]. Each locus, observed as a single band after electrophoresis, was purified with the AxyPrep-PCR Kit (Axygen^TM^) and sequenced by SeqXcel Inc. (CA, USA) using the M13 universal primers (Forward-TGTAAAACGACGGCCAGT and Reverse-CAGGAAACAGCTATGACC, 5’-3’). For loci *recA* and *pntA*, new primers were designed (Table 2), and used to amplify fragments as follows: 10 min at 96°C for initial denaturalization, followed by 35 cycles (1 min at 96°C, 1 min at 59°C, and 1 min at 72°C), 10 min at 72°C for the final extension, and maintenance at 4°C. The amplicons were then sequenced using the designed primers for *recA* and *pntA* (Table 2).

The sequences for each locus, were queried against the *V*. *parahaemolyticus* PubMLST database, to determine the allelic profile (AP) and sequence type (ST) (S1 Table). Novel alleles were submitted to PubMLST; the nucleotide sequences data that were reported for the MLST are available in the GenBank database under accession numbers KP455743-KP455966.

Haplotype diversity (Hd), pairwise nucleotide diversity (π) genetic variability (θ) and number of polymorphic sites (PSs), were determined for individual loci (S2 Table) using DnaSP, version 5.10.1 [30]. A neighbor-joining tree was constructed with concatenated sequences of these loci, with a final sequence of 3682 bp (in the following order: *dnaE*, *gyrB*, *recA*, *dtdS*, *pntA*, *pyrC* and *tnaA*), using Mega 5.2.2. [31], Kimura’s 2-parameter model and bootstrapping methods (1000 replicates).

The APs that were generated by MLST, were used to assign the strains to a clonal complex (S1 Table) with goeBURST, V1.2.1 (www.phyloviz.net/goeburst, [32]. The APs of the 32 strains were compared using Kimura’s 2-parameter model, against the database for *V*. *parahaemolyticus* strains in PubMLST, including within the CC3 [14, 33].

### Genomic analysis

Genomic DNA of an overnight LB culture of a single colony of the O3:K6 *V*. *parahaemolyticus* strain (CICESE-170), isolated in 1998, was extracted using the Wizard^TM^ Genomic kit (Promega). The quantity and quality of the DNA were determined on a NanoDrop^TM^ 2000 (Thermo Scientific, Wilmington, DE), and the DNA was diluted to ~1000 ng/μl in DNAse/RNAse-free water. Whole-genome sequencing was performed using Illumina Myseq™ genome analyzer (Illumina Inc., USA) per the manufacturer’s instructions.

The reads that were obtained from the CICESE-170 strain were assembled into contigs using CAP3 [34] and Vague 1.0.5 [35]. The synteny of contigs was obtained with Mauve 2.3.1 [36], using both chromosomes of the *V*. *parahaemolyticus* strain RIMD 2210633 (GenBank accession number: BA000031 and BA000032) as the reference genome [37].

Comparative analyses were performed between the whole-genome sequencing results on CICESE-170 and RIMD 2210633 as the reference strain. Contigs that were obtained from CICESE-170 were submitted and inspected with Rapid Annotation using Subsystem Technology (RAST) server (http://rast.nmpdr.org), and comparisons were made, based on the functions and sequences of both strains. The analyses were evaluated in the Seed Viewer, focusing on virulence, disease, defense, phages, prophages, transposables elements, plasmids, iron acquisition and stress response. Contigs from CICESE-170 were also inspected by alignment using Geneious 4.8 [38] and BLAST analysis to compare them with the reference strain. Whole-genome sequences of CICESE-170 were submitted to GenBank under accession number MIEM01000000.

## Results

### Species identification

In this study, the same *V*. *parahaemolyticus* strains were identified at the species level using the *tlh* or the pR72H primers; thus, both sets of primers could be used to determine species. Of the InDRE *V*. *parahaemolyticus* collection, 19 isolates were *tdh*^+^/*trh*^*-*^ and 18 were ORF8^+^. The 6 environmental strains that were isolated from biofouling were *tdh*^+^/*trh*^*-*^, 1 of which was confirmed to be ORF8^+^ (CICESE-273). The 4 isolates from the oyster samples were *tdh*^+^/*trh*^+^. From the CAIM collection, 8 strains were *tdh*^+^/*trh*^*-*^, 1 was *tdh*^+^/*trh*^*+*^ and 6 were ORF8^+^ as reported (Table 1).

Twenty-five ORF8^+^ strains were positive for the O3 and K6 antisera, including those that have been isolated by InDRE since 1998. The strains that were isolated during the 2004 outbreak in Mexico (CAIM collection) and 1 environmental strain from biofouling of a ship hull from 2012 (Table 1) were also ORF8^+^ and O3:K6^+^.

### PFGE analysis

**Fig 1:**
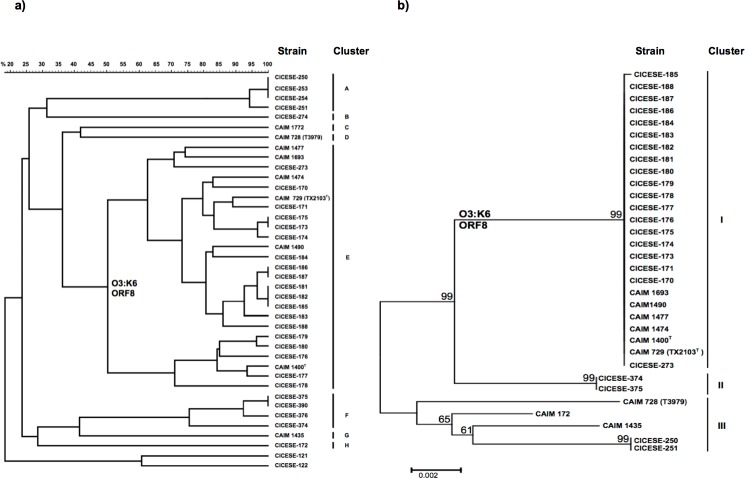
Dendrograms of CICESE and CAIM *V*. *parahaemolyticus* O3:K6 strains for: a) the neighbor-joining tree (UPGMA) by PFGE analysis after digestion with NotI and b) the neighbor-joining tree (Kimura’s 2 parameters) by MLST of the concatenated sequences of 7 loci.

Restriction fragments that were over 48.5 kb were examined, generating 2 branches (Fig 1A). Branch I contained all *V*. *parahaemolyticus* strains and branch II contained the out-group of *V*. *cholerae* strains. *V*. *parahaemolyticus* strains had high diversity, 31 patterns were obtained from 38 strains. At 50% of similarity, branch I split into 8 clusters (A to H), according to their genetic and serological characteristics (*tdh*^*+*^/*trh*^*+*^, *tdh*^*+*^ or *tdh*^*+*^/ORF8^*+*^/O3:K6). The lowest similarity among the 8 clusters was <24%.

The twenty-five O3:K6 strains, formed a single cluster (E), with >50% similarity and 21 PFGE patterns. Two clinical strains that were isolated in 1998 (CICESE-171 and CICESE-177) showed >88% and >93% of similarity with CAIM 729^T^ and CAIM 1400^T^, strains that were isolated during the Texas (USA) and Sinaloa (northwestern Mexico) outbreaks, respectively. Cluster E had low similarity (<37%) with the non-pandemic strains (clusters A, B, C, D, F, G, and H). Cluster A (n = 4) presented 2 PFGE patterns at >94% similarity; these strains (*tdh*^+^/*trh*^-^) were isolated from environmental samples (biofouling). Clusters B, D, G, and H contained 1 strain (*tdh*+ *trh*^-^), with less than a 29% similarity between clusters. Clusters C (n = 1) and F (n = 4) typified as *tdh*^+^/*trh*^+^, were isolated from environmental samples (shrimp and oysters).

### MLST analysis

The concatenated sequences of 7 loci that were analyzed from 32 selected strains of *V*. *parahaemolyticus* based on PFGE analysis, were separated them into 3 clusters according to their genetic and serological characteristics (Fig 1B). Cluster I comprised all the pandemic strains (*tdh*^*+*^/*ORF8*^*+*^/O3:K6), cluster II contained *tdh*^*+*^*/trh*^*+*^ strains, and cluster III was composed of *tdh*^*+*^ isolates. Cluster I included the strains that have been isolated by InDRE since 1998, those during the Mexican outbreak, a strain from the biofouling of a ship hull in provenance from Fukuyama, Japan (CICESE-273), and the pandemic strain (CAIM 729^T^), that was isolated during the Texas outbreak. With the exception of the CICESE-185 strain, all *ORF8*^*+*^/O3:K6 strains in cluster I had 100% similarity.

The 7 loci generated 43 alleles. Most loci had 6 alleles; the exception was *gyrB*, which had 7 (S1 Table). The *dnaE-3*, *recA-19*, *dtdS-4*, *pntA-29*, *pyrC-*4, and *tnaA-22* alleles were present in 25 strains, and *gyrB*-4 was presented in 24. Three loci had novel alleles due to the presence of a 1–2 bp difference and were assigned in the PubMLST database as *gyrB*-415, *recA*-295, and *tnaA*-231 (S1 Table).

The haplotype diversity (Hd) was 0.391 for most of the alleles; *gyrB* had an Hd of 0.44. The highest value for nucleotide diversity was observed for *recA* (π = 0.01054), whereas *pyrC had the lowest value* (π = 0.00378). The *recA* locus had the highest genetic variability (θ = 0.01345); *dnaE* had the lowest (θ = 0.00535). The number of polymorphic sites varied per locus, ranging from 11 for *dnaE* to 34 for *recA* (S2 Table).

The 5 novel STs that we obtained were submitted to the PubMLST database, assigned as ST-1137 (CAIM 1435), ST-1138 (CICESE-172), ST-1139 (CICESE-185), ST-1140 (CICESE-250 and 251), and ST-1141 (CICESE-374 and 375). All ORF8/O3:K6 strains contained ST-3, with the exception of strain CICESE-185, which harbored ST-1139; these strains were assigned to CC3 (S1 Table). The sequences that were obtained with the *recA* and *pntA* primers that we designed, had the same alleles (19 and 29) for all the O3:K6 strains, including the reference strain CAIM 729 (TX22103^T^), as previously reported with different primers [14].

**Fig 2:**
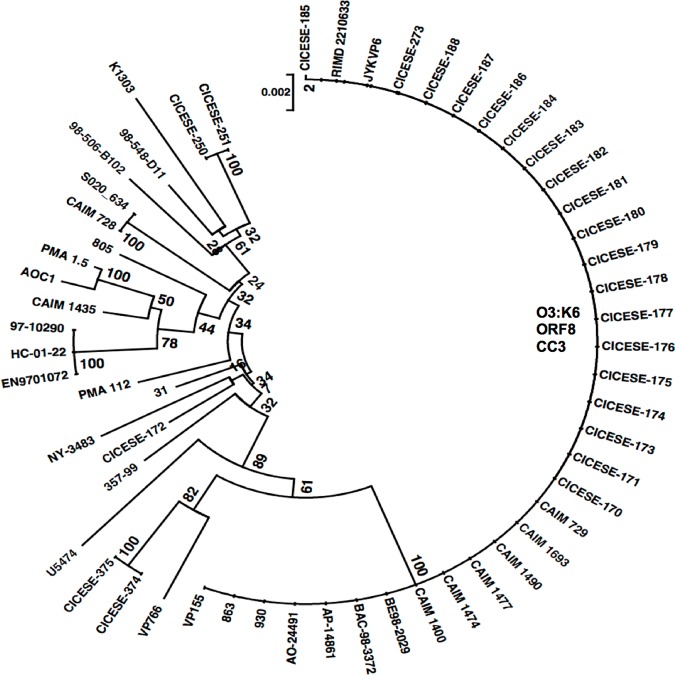
Assignment of CICESE/CAIM isolates and reference pubMLST sequences of O3:K6 and non-O3:K6 *V*. *parahaemolyticus* strains to a clonal complex.

The results on the 7 loci of concatenated sequences for 32 CICESE strains and 25 strains from pubMLST, are shown in Fig 2. Thirty-one strains from CICESE/CAIM had 100% similarity with the 9 strains from pubMLST that have been isolated worldwide, reported as O3:K6. Non-O3:K6 CICESE/CAIM and reference strains were grouped separately (Fig 2).

### Whole-genome analysis

Whole-genome sequencing of the CICESE-170 strain generated 1,970,230 (2 x 150 bp) paired-end reads that were assembled into 695 contings (N_50_ = 12,542 bp) at 52x coverage. The genome was 5,029,544 bp with a GC content of 45.3%. Analysis with the RAST server identified 4513 coding sequences (CDSs), 49 predicted RNAs, and 143 possibly missing genes. These CDSs were distributed into 26 categories, 104 subcategories, and 528 subsystems (RAST Job: 304198). The categories included virulence, disease, and defense (94 CDSs); iron acquisition system and metabolism (65 CDSs); stress response (173 CDSs); and phages, prophages (subsystem) (5 CDSs).

**Fig 3:**
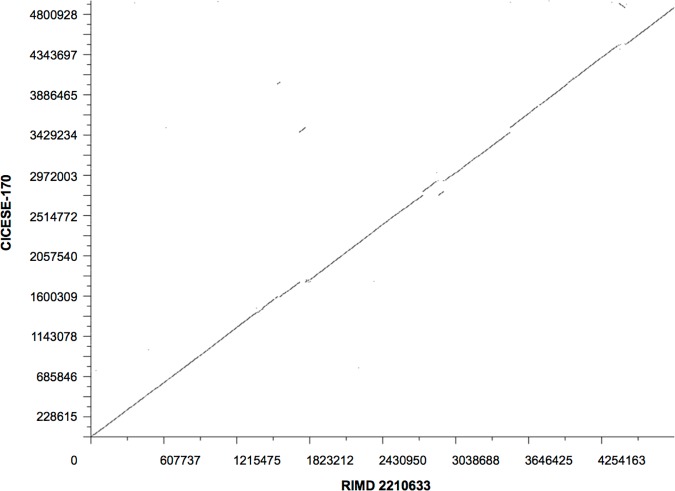
Whole-genome comparison of CICESE-170 and RIMD 2210633, implemented in BlastDotPlot.

A comparative analysis of concatenated contigs showed that the CICESE-170 and RIMD 2210663 strains are >99.3% similar (Fig 3).

The CICESE-170 strain contained 7 pathogenic islands (VPals), commonly described for *V*. *parahaemolyticus* O3:K6 pandemic strains (Table 3); 5 VPals (VPal-1 to VPal-5) lay in contigs that were associated with chromosome I, and 2 VPals (VPal-6 and VPal-7) resided in contigs of chromosome II. Most VPals (VPal-1 to -6) had higher that 99.7% similarity with RIMD 2210633 and had a GC content of 38.3% to 44.3%. VPal-7 contained the virulence factors, T3SS2 and the *tdh* gene, with 96.9% similarity to the reference sequence. Other elements in CICESE-170, commonly linked to the pandemic group, were the O- and K-antigen genes (VP0190-VP0238) and the phage f237 (VP1549-VP1562) with its corresponding ORF8 (VP1561). These elements were 100% similar to the reference sequence.

**Table 3:** Comparative results between CICESE-170 and RIMD 2210633.

	RIMD 2210633	CICESE-170			
Elements	*Location	CDS	bp	%GC	*similarity %
Vpal-1	VP0380-VP0403	24	22,380	41.5	100.0
Vpal-2	VP0635-VP0643	9	8286	44.3	99.9
Vpal-3	VP1071-VP1095	24	30,913	42.2	99.9
Vpal-4	VP2131-VP2144	14	16,160	38.9	99.9
Vpal-5	VP2900-VP2910	11	15,134	38.3	99.9
Vpal-6	VPA1253-VPA1270	18	26,750	43.4	99.7
Vpal-7 (T3SS2)	VPA1310-VPA1398	87	75,384	38.6	96.9
Phage f237	VP1549-1590	15	8,755	45.1	100
O3 and K6	VP0190-VP0238	49	51,483	40.4	100

* Based on results from *V*. *parahaemolyticus* RIMD 2210633.

## Discussion

Our results indicate that *V*. *parahaemolyticus* O3:K6 (*tdh*^+^/ORF8^+^) pandemic strains have been linked to clinical cases since 1998 in 6 Mexican states, particularly in the northwestern state of Sinaloa, most of which failed to develop into an outbreak [20]. Watery diarrhea, which is the most common symptom of *V*. *parahaemolyticus* infection, is self-limiting resolving in 3 days; thus, it is possible that most of O3:K6 infections were not reported or detected by the Mexican health system. Further, these reports might have been masked by the Mexican cholera epidemic from 1991 to 2000 [22].

In this study, by PFGE analysis, all strains with the characteristics of pandemic strains (*tdh*^+^/ORF8^+^/O3:K6) were grouped into a single cluster (E), with 21 patterns (Fig 1A). Wong *et al*. [9] and Yeung *et al*. [39] also assembled the pandemic strains into a single cluster with fewer PFGE patterns (8 and 7, respectively) between their O3:K6 isolates. Even if PFGE analysis reveals relative low similarity between pandemic strains, they are clearly separated from non-O3:K6 strains. PFGE results contrast with the high similarity among O3:K6 strains that is observed with other molecular approaches, such as MLST [14] and *toxRS* sequencing [40]. PFGE has been used frequently to discriminate between strains from various regions [41], but no such differentiation was noted in our study.

By MLST, using 7 housekeeping genes, Mexican *V*. *parahaemolyticus* isolates showed high genetic similarity between them. Most of the *tdh*^*+*^/ORF8^*+*^/O3:K6 strains isolated in Mexico, contained ST-3 and were thus associated with reference strains of the pandemic clone CC3 (Fig 2; S1 Table), which have been isolated worldwide since 1996, as did the reference strain CAIM 729 (TX22103^T^) [14, 33].

Notably, in 1998, seafood-associated clinical cases of *V*. *parahaemolyticus* O3:K6 were reported in Peru, Chile, and the US [15–17], the same year in which most InDRE strains were isolated. Thus, it appears that the O3:K6 pandemic clone encountered favorable conditions for its dispersion in these countries, including Mexico, likely due to the strong El Niño event that was registered in 1997–1998, as suggested [42].

Whole-genome sequencing of the CICESE-170 strain indicated a high genetic similarity with the reference sequence of RIMD 2210663 (>99.3%). Both strains shared mobile genetic elements, such as VPals (Table 3). RAST server analysis showed that CICESE-170 contained 4513 CDSs, 319 fewer than RIMD 2210663 (4832 CDSs). These differences might be associated with the adaptation to local environmental conditions, because similar disparities have been reported among O3:K6 strains from various countries [43].

All O3:K6 strains were positive for the ORF8 region by PCR, a region that is present in the phage f237 and was detected in CICESE-170, showing 100% similarity compared with RIMD 2210633 (Table 3). This phage has been used as a molecular marker due to its high specificity for the pandemic group [44] and according to Nasu *et al*. [45], it is involved in the epidemic potential of *V*. *parahaemolyticus* O3:K6.

Whole-genome sequencing showed that CICESE-170 contained 7 VPals that are common to *V*. *parahaemolyticus* O3:K6, most of which had high genetic similarity with the reference pandemic strain RIMD 2210633 (Table 3). The 7 VPals in CICESE-170 had a low GC content (38.3% to 44.3%), versus those of the overall genome (45.3%) and an earlier report (45.4%) [37, 46].

The four VPals that characterize the post-1996 pandemic strains were also detected in CICESE-170, confirming that this strain, with its high similarity to other Mexican O3:K6 isolates by MLST, is associated with pandemic *V*. *parahaemolyticus* O3:K6, that has been isolated worldwide [43]. VPals 1, 4, 5 and 6, which have been identified as a distinctive characteristic of O3:K6 pandemic strains and their serovariants, might have been acquired by horizontal gene transfer and might provide the pandemic strains with the ability to infect humans or to adapt to several environments [46]. VPal-7, which harbors the main pathogenic elements (*tdh* and T3SS2) of *V*. *parahaemolyticus*, was also detected in CICESE-170, but it had the lowest genetic similarity (96.9%) to RIMD 2210633.

## Conclusions

The *V*. *parahaemolyticus* O3:K6 strains that were isolated in Mexico, were grouped into a single cluster by PFGE and MLST (Fig 1A and 1B). They were associated with O3:K6 sequences in the PubMLST database and were related to CC3 (Fig 2). Bioinformatics analysis of the CICESE-170 genome, demonstrated high genetic similarity with the reference sequence of the RIMD 2210633 strain (Fig 3) and the characteristic elements of pandemic O3:K6 strains. These findings show that *V*. *parahaemolyticus* O3:K6 pandemic strains, have been detected in Mexico since 1998 and confirm their persistence in this country from 1998 to 2012, probably without undergoing significant genetic changes. Whole genome analysis of additional Mexican strains need to be performed to confirm this statement.

## Supporting Information

S1 Table Allelic profiles of 7 loci by MLST analysis. ST (sequence type), CC (clonal complex), D (double) and S (singleton).
(DOCX)

S2 Table Statistics of the 7 loci employed in the MLST analysis of *V*. *parahaemolyticus*. Hd (haplotype diversity), π (pairwise nucleotide diversity), Θ (genetic variability), PSs (polymorphic sites).
(DOCX)
