# Supplementary material for: Genetic Analysis of Vibrio parahaemolyticus O3:K6 Strains That Have Been Isolated in Mexico Since 1998
Source: PLoS One. 2017 Jan 18;12(1):e0169722. doi: 10.1371/journal.pone.0169722 (PMC5242489; doi:10.1371/journal.pone.0169722)
Supplement: S1 Table — ST (sequence type), CC (clonal complex), D (double) and S (singleton). (DOCX) [file pone.0169722.s001.docx]

**S1 Table. Allelic profile of 7 loci by MLST analysis.**

| **Cepa** | ***dnaE*** | ***gyrB*** | ***recA*** | ***dtdS*** | ***pntA*** | ***pyrC*** | ***tnaA*** | **ST** | **CC** |
| --- | --- | --- | --- | --- | --- | --- | --- | --- | --- |
| **CAIM 728** | **28** | **28** | **44** | **46** | **61** | **49** | **38** | **326** | **D** |
| **CAIM 729^T^** | **3** | **4** | **19** | **4** | **29** | **4** | **22** | **3** | **3** |
| **CAIM 1400^T^** | **3** | **4** | **19** | **4** | **29** | **4** | **22** | **3** | **3** |
| **CAIM 1435** | **45** | **336** | **143** | **7** | **14** | **46** | **20** | **1137** | **S** |
| **CAIM 1474** | **3** | **4** | **19** | **4** | **29** | **4** | **22** | **3** | **3** |
| **CAIM 1477** | **3** | **4** | **19** | **4** | **29** | **4** | **22** | **3** | **3** |
| **CAIM 1490** | **3** | **4** | **19** | **4** | **29** | **4** | **22** | **3** | **3** |
| **CAIM 1693** | **3** | **4** | **19** | **4** | **29** | **4** | **22** | **3** | **3** |
| **CICESE-170** | **3** | **4** | **19** | **4** | **29** | **4** | **22** | **3** | **3** |
| **CICESE-171** | **3** | **4** | **19** | **4** | **29** | **4** | **22** | **3** | **3** |
| **CICESE-172** | **10** | **159** | **31** | **88** | **26** | **109** | **57** | **1138** | **S** |
| **CICESE -173** | **3** | **4** | **19** | **4** | **29** | **4** | **22** | **3** | **3** |
| **CICESE -174** | **3** | **4** | **19** | **4** | **29** | **4** | **22** | **3** | **3** |
| **CICESE -175** | **3** | **4** | **19** | **4** | **29** | **4** | **22** | **3** | **3** |
| **CICESE -176** | **3** | **4** | **19** | **4** | **29** | **4** | **22** | **3** | **3** |
| **CICESE -177** | **3** | **4** | **19** | **4** | **29** | **4** | **22** | **3** | **3** |
| **CICESE -178** | **3** | **4** | **19** | **4** | **29** | **4** | **22** | **3** | **3** |
| **CICESE -179** | **3** | **4** | **19** | **4** | **29** | **4** | **22** | **3** | **3** |
| **CICESE -180** | **3** | **4** | **19** | **4** | **29** | **4** | **22** | **3** | **3** |
| **CICESE -181** | **3** | **4** | **19** | **4** | **29** | **4** | **22** | **3** | **3** |
| **CICESE -182** | **3** | **4** | **19** | **4** | **29** | **4** | **22** | **3** | **3** |
| **CICESE -183** | **3** | **4** | **19** | **4** | **29** | **4** | **22** | **3** | **3** |
| **CICESE -184** | **3** | **4** | **19** | **4** | **29** | **4** | **22** | **3** | **3** |
| **CICESE -185** | **3** | **415** | **19** | **4** | **29** | **4** | **22** | **1139** | **3** |
| **CICESE -186** | **3** | **4** | **19** | **4** | **29** | **4** | **22** | **3** | **3** |
| **CICESE -187** | **3** | **4** | **19** | **4** | **29** | **4** | **22** | **3** | **3** |
| **CICESE -188** | **3** | **4** | **19** | **4** | **29** | **4** | **22** | **3** | **3** |
| **CICESE -250** | **19** | **295** | **295** | **223** | **136** | **11** | **13** | **1140** | **S** |
| **CICESE -251** | **19** | **295** | **295** | **223** | **136** | **11** | **13** | **1140** | **S** |
| **CICESE -273** | **3** | **4** | **19** | **4** | **29** | **4** | **22** | **3** | **3** |
| **CICESE -374** | **51** | **57** | **75** | **353** | **45** | **78** | **231** | **1141** | **S** |
| **CICESE -375** | **51** | **57** | **75** | **353** | **45** | **78** | **231** | **1141** | **S** |

ST (sequence type), CC (clonal complex) D (double) and S (singleton).
