# Supplementary material for: Genetic Analysis of Vibrio parahaemolyticus O3:K6 Strains That Have Been Isolated in Mexico Since 1998
Source: PLoS One. 2017 Jan 18;12(1):e0169722. doi: 10.1371/journal.pone.0169722 (PMC5242489; doi:10.1371/journal.pone.0169722)
Supplement: S2 Table — Hd (haplotype diversity), π (pairwise nucleotide diversity), Θ (genetic variability), PSs (polymorphic sites). (DOCX) [file pone.0169722.s002.docx]

**S2 Table. Statistics of the 7 loci employed in the MLST analysis of *V. parahaemolyticus*.**

| ***Locus*** | **Alleles** | **Hd** | **π** | **Θ** | **PSs (%)** |
| --- | --- | --- | --- | --- | --- |
| ***dnaE*** | **6** | **0.391** | **0.00413** | **0.00535** | **11 (1.97)** |
| ***gyrB*** | **7** | **0.44** | **0.00546** | **0. 00923** | **22 (3.71)** |
| ***recA*** | **6** | **0.391** | **0.01054** | **0.01192** | **34 (4.66)** |
| ***dtdS*** | **6** | **0.381** | **0.00825** | **0.01345** | **25 (5.45)** |
| ***pntA*** | **6** | **0.391** | **0.00571** | **0.00808** | **14 (3.25)** |
| ***pyrC*** | **6** | **0.391** | **0.00378** | **0.00655** | **13 (2.63)** |
| ***tnaA*** | **6** | **0.391** | **0.00633** | **0.00822** | **14 (3.30)** |

Hd (haplotype diversity), π (pairwise nucleotide diversity), Θ (genetic variability), PSs (polymorphic sites).
